# An integrated evolutionary optimization and machine learning framework for predicting pharmacokinetics of nanocarrier-encapsulated drugs

**DOI:** 10.3389/fmed.2026.1965068

**Published:** 2026-09-30

**Authors:** Shaker T. Alsharif

**Affiliations:** Pharmaceutical Science Department, College of Pharmacy, Umm Al Qura University, Makkah, Saudi Arabia

**Keywords:** brain targeting ratio, multi-objective optimization, nanocarrier, pharmacokinetics, principal component analysis

## Abstract

This study developed a machine learning framework for predicting the pharmacokinetic behavior of nanocarrier systems using integrated molecular, physicochemical, and formulation descriptors. A Multilayer Perceptron Regressor (MLPR) was employed as the baseline predictive model, while evolutionary optimization algorithms were used to optimize the neural-network architecture and training parameters. The framework was evaluated for predicting three pharmacokinetic endpoints: maximum concentration (Cmax), time to maximum concentration (Tmax), and area under the concentration–time curve (AUC). The combined model produced 20%–30% reductions in RMSE for Cmax and AUC and kept *R*^2^ values above 0.85, while Tmax remained comparatively more challenging to model than Cmax and AUC, reflecting the stronger influence of dynamic absorption and release-related processes on temporal pharmacokinetic behavior. The results demonstrate that optimizer-guided neural-network tuning can improve pharmacokinetic prediction from heterogeneous nanocarrier datasets. The proposed framework provides an approach for improving the prediction of Cmax, Tmax, and AUC while explicitly considering predictive uncertainty and computational trade-offs.

## 1 Introduction

Nanocarrier drug delivery systems represent the next generation modalities to improve the pharmacokinetic performance of therapeutic agents. These agents are particularly those that are unstable, limited in membrane permeability, and have restricted access to the target tissue, such as the brain (1, 2). The current formulation may include a combination of variables such as particle size, surface charge, localization of the drug, encapsulation efficiency, shape, and presence of ligands on the surface, all of which have an impact on the drug release rate, absorption profile, and biodistribution (3–5). However, the connection between these physicochemical characteristics and pharmacokinetics to be observed *in vivo* is still ambiguous and cannot be predicted successfully (6–8). Key pharmacokinetic metrics to evaluate drug release, such as Cmax, Tmax, and AUC, are the most important parameters that indicate systemic exposure as well as delivery efficiency. Thus, Cmax depicts the highest concentration attained in plasma or the target tissue (9, 10); on the other hand, Tmax shows the time needed to reach that concentration, and thus, it is helpful in determining the absorption and release rates. AUC, which stands for the area under the curve of the drug concentration against time, is considered a comprehensive measure of total drug exposure and bioavailability (11). Altogether, these parameters explain in detail how formulation variables determine the kinetic profile of nanocarrier-mediated delivery. Conventional pharmaceutical formulation development primarily relies on empirical optimization strategies, including one-factor-at-a-time experimentation, factorial design, response surface methodology, and repeated *in vitro*/*in vivo* screening. Although these approaches have been widely adopted, they become increasingly inefficient for nanocarrier systems due to the large combinatorial design space involving particle size, surface charge, composition, drug loading, and release characteristics. Furthermore, conventional experimental approaches are often unable to efficiently capture nonlinear interactions between formulation descriptors and pharmacokinetic outcomes, thereby limiting generalizability across studies and slowing formulation optimization (12, 13).

This inconsistency in the results of nanocarrier-based experiments accounts for the materials, surface chemistry, administration routes, and biological milieus (14). The traditional empirical methods are unable to generalize these interactions across a wide range of formulations (15, 16). Therefore, the use of data-driven modeling as a tool to uncover patterns, streamline design, and forecast *in vivo* pharmacokinetic performance without any direct testing has become increasingly popular (17, 18). It is still very problematic to bring medications to the CNS because of the BBB, which is a very selective barrier. As the demand for targeted and efficient treatments of neurological disorders grows, research is more and more directed towards the use of nanocarrier systems that can facilitate drug penetration into the brain and can also be used for improving PK behavior (19–21). In general, by far most of the works profoundly concentrate on biological ways or carrier construction; however, the quantitative modelling of PK changes for different nanocarrier parameter sets to different biological outputs is still vague (22).

### 1.1 Nanocarrier and BBB-targeting strategies in existing research

Different strategies to bypass or change the BBB have been the subject of extensive research. A number of reviews shed light on the physiological barriers of the BBB and assess nanoparticle-based delivery systems, such as polymeric nanocarriers, ligand-functionalized carriers, and micellar structures, as potential vehicles for improved brain delivery (23–25). Besides that, the research has been done on stem-cell-based carriers, the mesenchymal stem cells in particular, which may have the extraordinary ability to deliver the therapeutic cargos across the BBB via the natural cellular transport mechanisms (26). Lipid nanoparticles are turning out to be the most attractive delivery systems as well. By surface-modifying the structures, it is possible to achieve more efficient BBB penetration and then apply it to brain cancer therapy (27). In parallel, there are also initiatives to open the BBB with the help of devices that use ablative or modulation technologies and can show transient permeability and hence better drug distribution in brain tissues (28). Non-invasive delivery routes, such as nasal administration, glymphatic-system-based transport, and alternative CNS access routes, which are beyond direct BBB targeting, also give the possibility to go around the BBB restrictions (29, 30). At last, thorough reviews summon up the current difficulties in CNS drug delivery and put forward biological, chemical, and physical means for trans-BBB transport enhancement, which indicates that the field is still more focused on the mechanistic understanding than on the quantitative prediction (31, 32).

### 1.2 Limitations of prior literature and current predictive gaps

While studies on such topics have come a long way, these have mostly been qualitative or mechanistic in nature (23–25). They have failed to put forward a quantitative relationship between specific physicochemical properties of nanocarriers, like size, surface charge, shape, and encapsulation efficiency, and essential aspects of PK such as Cmax, Tmax, and AUC. Besides, they are missing the level of cross-study data integration needed for drawing generalizable conclusions, although large datasets (e.g., 403 × 24 design matrices derived from 237 studies) are already available. In addition, none of the reviewed works applies ML models such as multilayer perceptron regressors to directly infer PK behaviour from formulation features, nor do they use PCA, k-fold validation, Friedman statistical ranking, or prediction-interval bootstrapping. Multi-objective instruments like Pareto-front analysis are also not present in the current methods. Despite substantial advances in nanocarrier engineering, formulation selection remains heavily dependent on labor-intensive experimentation and fragmented literature evidence. There remains a practical need for predictive computational frameworks capable of estimating pharmacokinetic performance directly from formulation descriptors prior to synthesis or animal testing. Such models could accelerate formulation prioritization, reduce development costs, and support more rational nanomedicine design.

### 1.3 Contribution of the present study

This study proposes a machine learning framework for predicting pharmacokinetic endpoints (Cmax, Tmax, and AUC) directly from nanocarrier physicochemical descriptors using an integrated multi-study dataset derived from published *in vivo* investigations. The principal contribution of this study is not the introduction of a new machine learning algorithm, but the development of an integrated computational workflow for pharmacokinetic prediction from heterogeneous literature-derived nanocarrier datasets. The framework combines nonlinear regression, evolutionary hyperparameter optimization, bootstrap-based uncertainty quantification, repeated validation, and multi-objective model assessment within a unified pipeline specifically designed for cross-study pharmacokinetic prediction. To the best of current knowledge, such an integrated workflow has not previously been reported for prediction of nanocarrier pharmacokinetic endpoints using heterogeneous multi-study formulation descriptors.

## 2 Dataset and preprocessing methods

A multi-study dataset for predictive modelling of nanocarrier pharmacokinetics was compiled by performing a structured literature search in the PubMed and ScienceDirect databases. A search yielded 237 qualifying research articles, which report *in vivo* experiments of nanocarriers administered via the nasal route with a comparative reference route, mainly intravenous (33). Combining data from several independent studies offers a broad and biologically relevant basis for the development of a generalizable predictive framework. The literature-derived dataset did not explicitly encode drug identity as a standalone categorical variable. Instead, drug-specific characteristics were represented through physicochemical descriptors including molecular weight, lipophilicity (logP), solubility, pKa, and P-glycoprotein substrate status, thereby preserving molecular information while avoiding excessive categorical sparsity in the integrated multi-study dataset.

### 2.1 Inclusion and exclusion criteria

The studies considered for inclusion were only those that satisfied the predetermined criteria, which guarantee the consistent and complete reporting of both the characteristics of the formulations and pharmacokinetic measurements. Eligible publications had to:

- Description of nanoparticle-based formulations for nasal administration with IV data for comparison;
- Provide physicochemical descriptors that include particle size, surface charge, encapsulation efficiency, drug localization (core or shell), particle shape, and surface ligands or modifications;
- Report release-study data together with the relevant experimental variables, e.g., exposure time and drug accumulation;
- Present at least one pharmacokinetic outcome in both plasma and brain, specifically Cmax (ng/mL), Tmax (h), or AUC (ng·h/mL), allowing the calculation of ratios such as AUC_brain/AUC_plasma. Studies missing essential formulation attributes or PK endpoints were excluded in order to maintain the integrity of the dataset.

The final dataset consisted of 403 unique experimental observations extracted from 237 literature sources. Multiple observations from individual studies were retained only when corresponding to distinct formulations, administration conditions, or experimentally reported pharmacokinetic measurements. Therefore, the final sample count reflects observation-level granularity rather than study-level duplication.

### 2.2 Data cleaning and feature engineering

Preparation activities included the dropping of incomplete records, the handling of missing values in a consistent way, and the checking for outliers in order to have numerical ranges that are consistent. The encoding of categorical descriptors was done in an appropriate manner, and the standardization of continuous variables was also performed in order to avoid scale-driven bias in model training. All these steps made it possible to compare different studies that come from different labs. In order to build the models, data was divided into training and validation sets through k-fold cross-validation, ensuring the models' generalization power and reducing the risk of data leakage. Principal component analysis was introduced primarily to reduce multicollinearity among correlated physicochemical descriptors, improve numerical conditioning, and reduce redundancy before neural network optimization. It was not intended to eliminate heterogeneity arising from integration of multiple studies, since inter-study variability remains inherently present after linear transformation. Instead, PCA served as a preprocessing strategy to produce a more compact feature representation and improve optimization stability. Although preprocessing strategies such as normalization and dimensionality reduction were implemented to reduce scale-driven variance, residual heterogeneity originating from literature aggregation cannot be fully removed. To mitigate overfitting risk, the framework incorporated repeated K-fold validation, uncertainty quantification through bootstrapped prediction intervals, and optimizer stability assessment. Records containing missing values in essential pharmacokinetic endpoints or critical formulation descriptors were excluded from model development because reliable imputation was not considered appropriate for heterogeneous literature-derived measurements. Binary categorical variables (e.g., P-glycoprotein status) were encoded as 0/1, whereas categorical formulation descriptors with multiple categories were represented using integer encoding according to predefined formulation classes. Continuous variables were standardized to zero mean and unit variance before PCA to eliminate scale-dependent bias. Principal component transformation was subsequently performed using only standardized predictor variables before model training.

### 2.3 Multivariate interaction topography of formulation variables and pharmacokinetic outputs

Figure 1 visualizes the interaction patterns between the main physicochemical variables of the formulation and the pharmacokinetic outputs (Cmax, Tmax, and AUC), which serve as a first nonlinearity and multivariate diagnostic of the dataset. Several significant trends can be identified from the contour surfaces. Gradients of considerable magnitude can be seen in formulation weight, solubility, and the drug-to-carrier ratio for Cmax, implying that these parameters are the ones most tightly linked to changes in the initial concentration peak. In contrast, Tmax shows larger and less clearly defined transitions that suggest its temporal changes are a result of the combined effects of particle size, surface charge, and hydrophobicity, and thus not attributable to a single factor only. The AUC topographies, by comparison, exhibit gentler but still quite distinct bright areas, which can be taken as an indication that overall drug exposure is more dependent on integrated formulation patterns than on individual variables. Individually, these heterogeneous and non-separable contour structures strengthen the notion of weak linear relationships that have been detected by the exploratory analysis and point to the presence of intricate interaction effects among the predictors. The behavior exhibited here calls for nonlinear and interaction-aware modeling frameworks; hence, MLPR, evolutionary optimizers, and uncertainty-based evaluation strategies are expected to be used in the next modeling phases.

**Figure 1:**
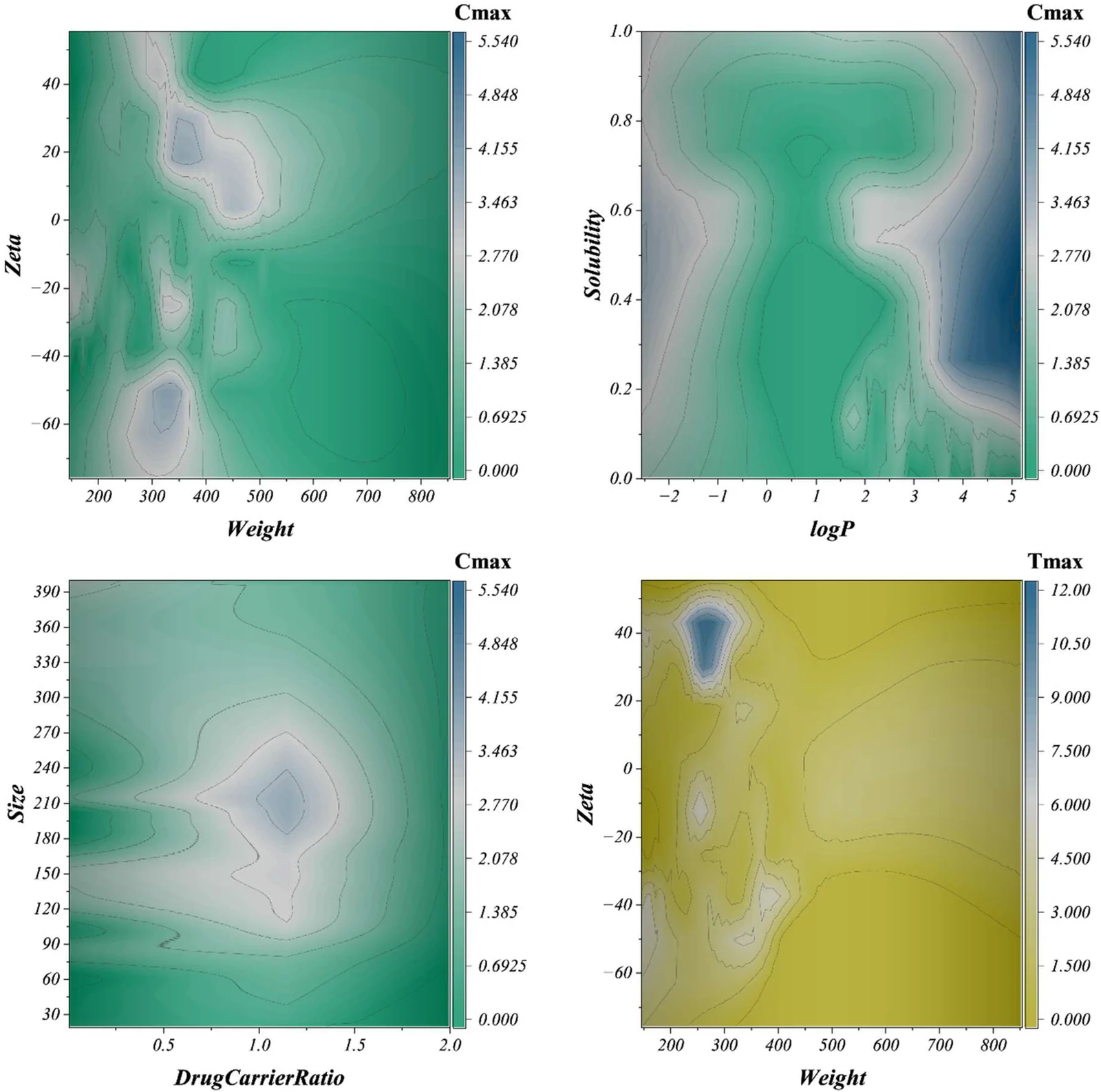

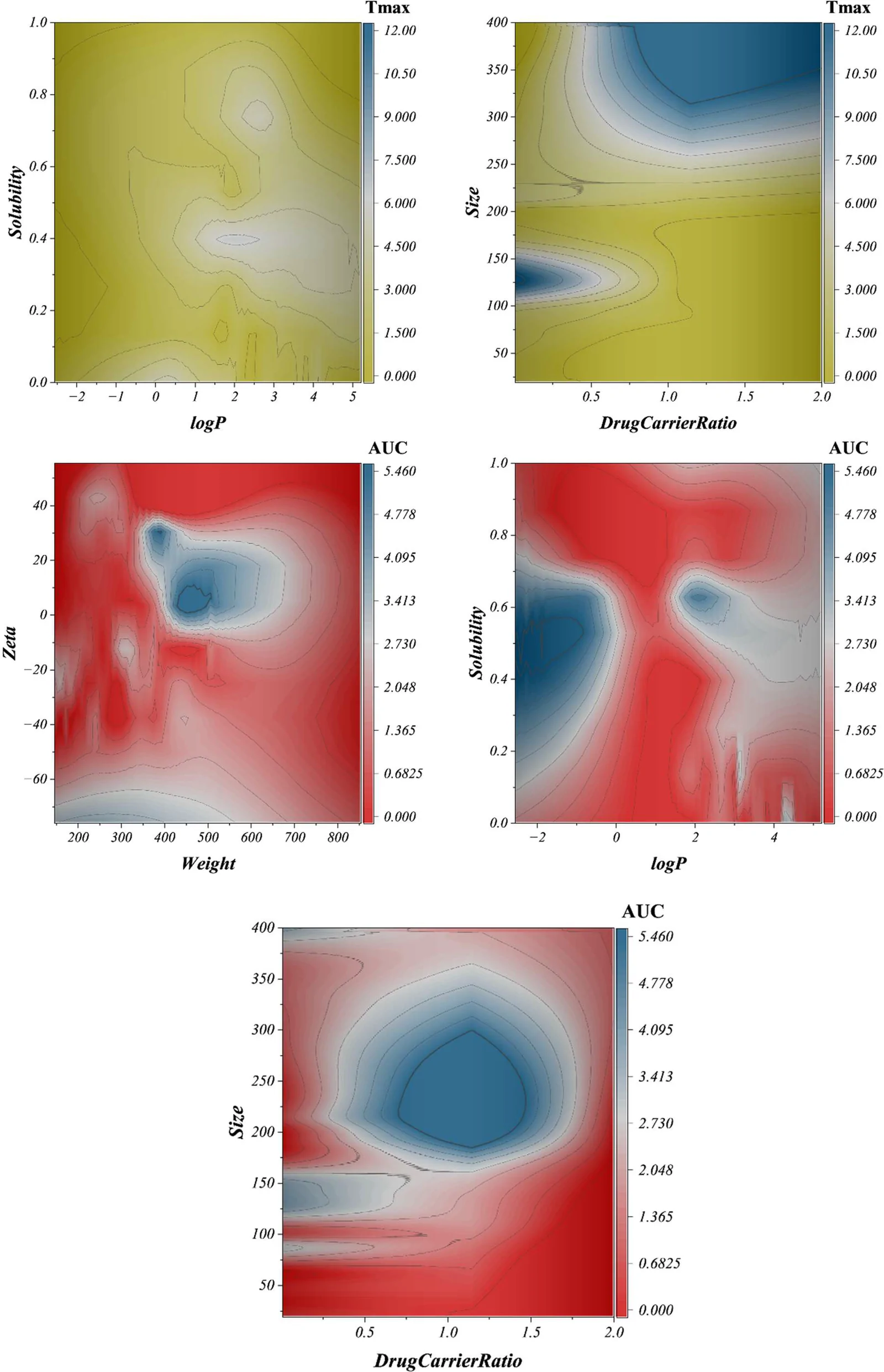
Contour plot for the normal features and the target variable, providing a clear visualization of their pairwise relationships, distribution patterns, and potential interdependencies.

### 2.4 Descriptive statistics highlighting dataset heterogeneity and cross-study variability

Table 1 summarizes the statistical properties of both categorical and continuous variables, allowing an understanding of the dataset's heterogeneity and thereby informing the modeling strategies that were applied. The categorical descriptors of the formulation (e.g., P-gp status, drug position, composition types, and administration route) are evenly distributed across their respective ranges. Such a balanced representation practically excludes the risk of bias driven by categories and also guarantees that no single class will exert its influence on the learning process disproportionately. On the other hand, the continuous physicochemical variables reveal wide numerical ranges, large variances, and asymmetric distribution patterns. Thus, the parameters such as particle weight, size, zeta potential, and drug-to-carrier ratio have very large standard deviations and wide dynamic spans, while the pharmacokinetic outputs (Cmax, Tmax, AUC) are similarly heterogeneously distributed. The reason for such variability is the combination of data from different studies and various experimental conditions, thus highlighting the cross-study diversity present in the dataset. In essence, these statistical features collectively signify that the data have heteroscedasticity, as well as most likely nonlinear, interaction-dependent relationships among the predictors. Hence, the use of dimensionality reduction (PCA) to handle mixed-scale variability, nonlinear modeling through MLPR to capture complex dependencies, evolutionary optimizers to find their way through irregular error surfaces, and bootstrapped prediction intervals to indicate uncertainty due to heterogeneous variance is justified.

**Table 1:** Assessment of input variables and the target variable to support robust model development.

Distribution	Variable	Indicator					
		Lower bound		Upper bound		Mode	
Uniform	Pgp	0		1		1	
	Position	1		2		1	
	Comp1	1		10		5	
	Comp2	1		10		8	
	Route	1		2		1	
		Min.	Max.	Avg.	Median	MAD.	St. Dev.
Normal	Weight	146.14	853.91	319.05	315.03	68.032	92.621
	logP	−2.56	5.21	2.769	2.93	0.968	1.234
	Solubility	0	1	0.159	0.071	0.154	0.220
	DrugCarrierRatio	0.007	2	0.285	0.1	0.253	0.343
	Size	19	400	147.69	150.2	52.99	71.460
	Zeta	−76	55.47	−8.941	−15.78	19.443	25.281
	Cmax (ng/mL)	0.008	5.522	1.112	0.882	0.845	1.071
	Tmax (h)	0.028	12	2.257	1	1.782	2.277
	AUC (ng·h/mL)	0.01	5.456	1.142	0.813	0.886	1.128

### 2.5 Principal component analysis revealing global variance structure and mixed-scale predictors

The integrated dataset contains predictors with substantially different numerical scales, distributions, and variance magnitudes, including continuous molecular descriptors, physicochemical variables, and categorical formulation parameters. Variance structure analysis was therefore performed to examine feature dispersion patterns, scale dominance, and potential redundancy prior to model training. As shown in Table 2, particle size and molecular weight exhibited substantially larger variance magnitudes than several lower-scale descriptors such as logP, solubility, and drug-carrier ratio. This observation reflects the mixed-scale nature of the dataset and highlights the importance of normalization and scale-aware preprocessing to prevent high-dispersion variables from dominating model training. Lower-variance predictors were retained despite their smaller global variance contribution because reduced variance does not imply reduced predictive relevance, particularly in nonlinear learning settings where interaction effects and localized relationships may remain highly informative. The variance structure analysis therefore primarily served as a preprocessing diagnostic rather than a biological ranking procedure.

**Table 2:** Variance structure summary of mixed-scale predictors used to assess feature dispersion and preprocessing requirements.

Variable	Parameter		
	Explained variance ratio	Explained variance	Singular value contribution
Weight	6.3 × 10^−1^	8.6 × 10^3^	1.1 × 10^3^
logP	5.6 × 10^−6^	7.7 × 10^−2^	3.7 × 10
Solubility	1.9 × 10^−7^	2.7 × 10^−3^	6.4 × 10^−−1^
Pgp	8.2 × 10^−7^	1.1 × 10^−2^	1.7 × 10
DrugCarrierRatio	6.3 × 10^−8^	8.7 × 10^−4^	4.8 × 10^−1^
Position	2.3 × 10^−7^	3.1 × 10^−3^	8.7 × 10^−1^
Comp1	4.0 × 10^−5^	5.5 × 10^−1^	1.1 × 10^1^
Comp2	4.7 × 10^−7^	6.5 × 10^−3^	1.1 × 10
Size	3.7 × 10^−1^	5.1 × 10^3^	8.9 × 10^2^
Zeta	3.0 × 10^−3^	4.2 × 10^1^	7.9 × 10^1^
Route	7.0 × 10^−8^	9.7 × 10^−−4^	5.0 × 10^−−1^

### 2.6 K-Fold cross-validation performance of MLP models across pharmacokinetic endpoints

Table 3 summarizes the five-fold cross-validation performance of the MLPR across Cmax, Tmax, and AUC. Cmax and AUC showed comparatively stable performance across the validation folds, with consistently strong *R*^2^ values and relatively limited RMSE variation. In contrast, Tmax exhibited greater fold-to-fold variability, indicating that this temporally dependent endpoint was more difficult to predict consistently. Overall, the results suggest that Cmax and AUC were more stable modeling targets, whereas Tmax was more sensitive to variations in the training and validation partitions.

**Table 3:** Results generated from the applied K-fold procedure.

Model	Target	Indicator	Number of K-Folds				
			*K1*	*K2*	*K3*	*K4*	*K5*
MLP	*Cmax*	RMSE	0.362	0.312	0.355	0.316	0.289
		*R* ^2^	0.848	0.945	0.859	0.923	0.953
	*Tmax*	RMSE	0.891	0.644	0.668	0.881	0.742
		*R* ^2^	0.762	0.949	0.939	0.760	0.880
	*AUC*	RMSE	0.356	0.350	0.408	0.284	0.256
		*R* ^2^	0.847	0.855	0.767	0.947	0.956

## 3 Methodology

This research outlines an integrated ML framework that incorporates a nonlinear predictive model, three advanced metaheuristic optimizers, and robust statistical validation techniques. The methodological pipeline is fivefold: data preprocessing, model training, optimization, performance evaluation, and multi-criteria decision analysis (34).

### 3.1 Predictive model: multilayer perceptron regressor (MLPR)

The Multilayer Perceptron Regressor (MLPR) was used as the baseline nonlinear predictive model because of its ability to approximate complex relationships among molecular, physicochemical, and formulation descriptors. The baseline model is referred to as MLPR throughout this manuscript. Optimizer-enhanced variants are identified according to the optimization algorithm used. Specifically, MLPR optimized using the Attack–Leave Optimizer is referred to as MLAL, whereas MLPR optimized using the Dynamic Arithmetic Optimization Algorithm is referred to as MLDA (35, 36). Multilayer perceptron regression was implemented as the primary nonlinear predictive model due to its ability to approximate complex multivariate relationships among molecular, physicochemical, and formulation descriptors. Network architecture parameters, including hidden layer configuration, neuron allocation, and training-related hyperparameters, were optimized using population-based search strategies. Candidate architectures were evaluated under repeated five-fold cross-validation, and final model selection was based on predictive accuracy, robustness, and uncertainty performance.

The Multilayer Perceptron Regressor (MLPR) was used as the primary nonlinear predictive model because of its ability to approximate complex multivariate relationships among molecular, physicochemical, and formulation descriptors. The network consisted of an input layer receiving the preprocessed predictor representation, two hidden layers with nonlinear hyperbolic tangent (tanh) activation functions, and a single-node linear output layer corresponding to each pharmacokinetic endpoint. The number of neurons in the two hidden layers was independently optimized for each pharmacokinetic endpoint and optimization algorithm. Therefore, a fixed network size was not imposed across Cmax, Tmax, and AUC. Candidate architectures were evaluated through repeated five-fold cross-validation, and the final configuration was selected according to cross-validated predictive performance. The optimized neuron allocations for each endpoint are reported in Table 4.

**Table 4:** Optimal training parameters of the proposed framework.

Parameter	Value
Prediction model	Multilayer Perceptron Regressor
Hidden layer architecture	Optimized separately for each PK endpoint
Activation function	Hyperbolic tangent (tanh)
Output activation	Linear
Learning rate	Optimized by evolutionary algorithms
Regularization coefficient (*α*)	Optimized
Optimizer	DAOA/ALO/OOA
Population size	30
Maximum optimization iterations	200
Fitness function	RMSE
Cross-validation	Repeated 5-fold
PCA preprocessing	Applied before training
Input normalization	Standardization
Prediction uncertainty	Bootstrap prediction intervals (95%)
Statistical comparison	Friedman + Wilcoxon tests

### 3.2 Optimization algorithms

The three population-based algorithms (DAOA, ALO, and OOA) were used to optimize the MLPR hyperparameters, which also included the number of neurons, learning rate, and regularization terms. Each Optimizer independently trained the MLPR, which allowed the comparative evaluation of their convergence behaviour and overall performance. DAOA, ALO, and OOA were selected because they represent distinct search dynamics and optimization philosophies, enabling comparative evaluation of exploration-exploitation balance, convergence behavior, and hyperparameter search robustness within a common predictive framework. Their inclusion was intended to assess optimizer sensitivity under nonlinear pharmacokinetic modeling conditions rather than to provide an exhaustive benchmark of all optimization strategies.

The optimized multilayer perceptron architecture was independently identified for each pharmacokinetic endpoint through evolutionary hyperparameter optimization. Network topology, learning parameters, and regularization coefficients were simultaneously optimized by minimizing cross-validated RMSE under repeated five-fold validation. The resulting optimal training configuration is summarized in Table 4 to facilitate reproducibility.

The optimization process simultaneously explored the number of neurons in the first and second hidden layers, the learning rate, and the regularization coefficient (*α*). The number of neurons in each hidden layer was searched within the range of 50–200 neurons. This range provided sufficient flexibility for identifying endpoint-specific network architectures while avoiding excessively large configurations. The learning rate was optimized within the range of 0.0001–0.01, covering conservative to moderately aggressive learning conditions. The regularization coefficient (*α*) was searched between 0.00001 and 0.01 to allow different levels of weight regularization. All candidate solutions were constrained within these predefined bounds during the population-based search. Each candidate MLPR configuration was evaluated using five-fold cross-validation, and the mean validation RMSE across the five folds was used as the optimization objective. The final hyperparameter configuration was selected according to the minimum cross-validated RMSE.

#### 3.2.1 Dynamic arithmetic optimization algorithm (DAOA)

The Dynamic Arithmetic Optimization Algorithm extends the basic arithmetic optimization framework by adding adaptive features that improve both the exploration and exploitation phases during the whole process of the training (37). Equations 1–3 presents mathematical formulas of used methods.

- *Dynamic Accelerated Function (DAF)*

The DAF controls the change from global exploration to local exploitation by means of a gradually decreasing function:

$$DAF={\left(\frac{Iter}{Iter_{\max}}\right)}^{\alpha }$$ (1)

This formulation ensures broad search capability during early iterations while gradually focusing the search as the algorithm approaches convergence (38).

- *Dynamic Candidate Solution (DCS)*

The operations of DCS represent a local search that is being continuously improved by limiting the range of the candidate solution with each iteration in accordance with:

DCS(t+1)=0.99×DCS(t) (2)

Such regulation of the search neighborhood encourages delicate adjustments to the solution space around the most promising regions (39). By and large, DAOA is a very parameter-efficient algorithm that still retains a high degree of flexibility and thus is capable of effectively dealing with the complicated nonlinear landscape that goes along with MLPR training.

#### 3.2.2 Attack–leave optimizer (ALO)

The Attack–Leave Optimizer achieves this balance through a multiphase attack plan that carefully balances the aspects of exploration and exploitation in the phases (40).

- *Phase 1: Directed Search I (exploitation):*

Basically, the candidate solutions in this first phase are to be brought, merely in some cases a bit beyond, the current global best. This direct motion strongly points to the exploitation concept by deepening the search in the closest neighbourhood of the already most promising region.

- *Phase 2: Directed Search II (hybrid exploration-exploitation):*

The second phase merges the data of several population members with the global best solution. This hybrid device is still a balancing act between diversification and intensification, which thus allows the algorithm to probe the new areas and at the same time not lose track of the guidance of high-quality solutions guidance.

- *Phase 3: Random Search (exploration fallback):*

This part of the program is about inserting randomness into the search to allow the algorithm to get out of local minima. By bringing back random moves, diversity is replenished, and the premature convergence is prevented by the algorithm. ALO also utilizes a very strict acceptance criterion whereby a new candidate solution is the one to replace the old one only if it brings about an improvement of the objective function. This method is an instrument for ensuring stable convergence, and thus, it keeps away from the unnecessary solution-space oscillations.

#### 3.2.3 Osprey optimization algorithm (OOA)

OOA simulates the behavior of an osprey hunting, and has two phases (41):

- *Phase 1: Locating/Hunting (exploration):*

Ospreys locate “fish” (better solutions) and quickly move in their direction:

$$x_{i,j}^{P1}=x_{i,j}+r_{i,j}(SF_{i,j}-I_{i,j}x_{i,j})$$ (3)

- *Phase 2: Fishing/Grasping (exploitation):*

The position is adjusted closely to the best one while still honoring the boundary constraints.

OOA starts with energetic exploration and ends with gradual convergence in the subsequent iterations (42, 43).

### 3.3 Model training and validation

Generalization performance was evaluated through repeated five-fold cross-validation, comparison of training and testing prediction errors, bootstrap-based uncertainty estimation, and optimizer stability analysis. Similar performance between training and testing datasets together with narrow prediction intervals indicated limited evidence of overfitting. Nevertheless, because the available literature-derived dataset was used for both model development and internal validation, complete assessment of model generalizability will ultimately require independent prospective validation using external pharmacokinetic datasets collected under different experimental conditions. Statistical differences among the three population-based optimization algorithms were examined using the Friedman rank test followed by Wilcoxon signed-rank tests (*p* < 0.05). Bootstrap resampling was additionally employed to estimate 95% prediction intervals and quantify predictive uncertainty without distributional assumptions. Model selection was further supported through Pareto-front multi-objective analysis, balancing predictive accuracy, computational efficiency, convergence stability, and robustness. Performance was comprehensively assessed using RMSE, MAE, *R*^2^, U95, and MAAPE, providing complementary measures of prediction accuracy, variance explanation, uncertainty, and numerical robustness.

The maximum number of 200 iterations refers to the population-based hyperparameter optimization process rather than continuous unrestricted neural network training. Candidate MLP configurations generated during optimization were evaluated using repeated five-fold cross-validation, and the optimal model was selected based on cross-validated predictive performance. Consequently, models exhibiting poor generalization across validation folds were not retained. In addition, bootstrap-based uncertainty estimation and comparison of training and testing performance were used to assess model robustness and identify potential overfitting. Although an explicit early stopping criterion was not implemented, the combined validation framework provided practical safeguards against overfitting. Future studies may further strengthen the training protocol through the incorporation of early stopping or nested cross-validation strategies.

A five-fold cross-validation strategy (*k* = 5) was used during model development and hyperparameter optimization. The available dataset was divided into five approximately equal subsets. For each cross-validation cycle, four folds were used for model training and the remaining fold was used for validation. This procedure was repeated five times so that each subset served once as the validation fold. During population-based optimization, each candidate MLPR configuration was evaluated using the five-fold procedure. The predictive performance obtained across the five validation folds was aggregated, and the mean RMSE was used as the objective function for selecting improved hyperparameter configurations. The fold-level RMSE and *R*^2^ values were also retained to assess the stability of model performance across different data partitions. After the optimal configuration was identified, the selected model was evaluated using the predefined training and testing procedure, while the cross-validation results were used as an additional assessment of robustness and generalization.

## 4 Results and discussion

### 4.1 Optimizer convergence and hyperparameter configurations for PK prediction

Table 5 summarizes the optimized architectures and convergence performance obtained for the pharmacokinetic endpoints. Both optimization algorithms converged within the maximum limit of 200 iterations and identified different neural-network configurations for each prediction task. The optimized architectures varied across pharmacokinetic endpoints. For Cmax, the Attack–Leave Optimizer and Dynamic Arithmetic Optimization Algorithm identified hidden-layer configurations of (138, 152) and (163, 129), respectively. For Tmax, the corresponding configurations were (112, 146) and (159, 133). For AUC, the optimized configurations were (162, 119) and (157, 174). The larger differences observed for Tmax indicate greater sensitivity of this endpoint to hyperparameter selection. In contrast, Cmax and AUC produced more comparable optimized architectures. These differences should be interpreted as characteristics of the optimization results rather than evidence that a specific pharmacokinetic endpoint inherently requires a particular neural-network structure.

**Table 5:** Optimized MLP architectures, convergence performance (RMSE), and hyperparameter configurations obtained by the population-based optimization algorithms for each pharmacokinetic endpoint.

Models	Target	Optimizers	Convergence of 200 Iterations (RMSE)	Optimized Hyperparameters	
*MLP*	*Cmax*	*Attack-Leave Optimizer*	Min = 0.248	Neuron	138
			Mean = 0.337	Neuron	152
			Max = 0.520		
		*Dynamic Arithmetic Optimization Algorithm*	Min = 0.196	Neuron	163
			Mean = 0.283	Neuron	129
			Max = 0.542		
	*Tmax*	*Attack-Leave Optimizer*	Min = 0.520	Neuron	112
			Mean = 0.693	Neuron	146
			Max = 1.168		
		*Dynamic Arithmetic Optimization Algorithm*	Min = 0.452	Neuron	159
			Mean = 0.697	Neuron	133
			Max = 1.247		
	*AUC*	*Attack-Leave Optimizer*	Min = 0.217	Neuron	162
			Mean = 0.291	Neuron	119
			Max = 0.481		
		*Dynamic Arithmetic Optimization Algorithm*	Min = 0.157	Neuron	157
			Mean = 0.232	Neuron	174
			Max = 0.422		

### 4.2 Comparative predictive performance of baseline and optimizer-enhanced MLP models

Table 6 compares the baseline Multilayer Perceptron Regressor (MLPR) with the optimizer-enhanced variants, including the MLPR optimized using the Attack–Leave Optimizer (MLAL) and the MLPR optimized using the Dynamic Arithmetic Optimization Algorithm (MLDA), across the three pharmacokinetic endpoints. In sum, the findings reveal that the incorporation of evolutionary optimizers into the model-training process leads to consistent improvements in accuracy, robustness, and prediction-interval precision. Regarding Cmax, the MLDA model was the most effective, leading to the lowest test RMSE and the highest *R*^2^ across all configurations tested. These enhancements imply that MLDA is the model that best explains the nonlinear interactions between nanocarrier formulation parameters and peak systemic exposure. Additionally, the shortened U95 interval is a signal of more stable and trustworthy predictions, thus reflecting the bootstrapped uncertainty bands in width. As for Tmax, all the models showed comparably higher error metrics, which is consistent with the increased physiological variability of the timing-based PK parameters. However, MLDA was once again the model with the highest predictive power, as it showed improvements both in RMSE and *R*^2^ when compared to the baseline MLP. The lower MAAPE values of the hybrid models are a sign of better resistance to the skewness or outlier of the Tmax observations, while the decrease of the U95 indicates that the optimizer-driven tuning enhances the reliability of the interval even for the more variable targets. Regarding AUC, the hybrid models were the main contributors to the performance increase. MLDA not only achieved a high level of training fidelity but also strong test generalization as evidenced by its low RMSE and high *R*^2^. The reduction in U95 in comparison to the single MLP implies that the optimizer-enhanced architectures provide more confident predictions of the overall drug exposure, a variable that is tightly linked to therapeutic efficacy.

**Table 6:** Key statistical measures for evaluating model performance, with emphasis on predictive accuracy and overall efficiency.

Target	Process	*Category*	ML Models	Evaluation Metrics				
				RMSE	*R* ^2^	MAE	U95	MAAPE
Cmax	Train	*Single Model*	MLP	0.305	0.957	0.174	0.833	0.230
		*Hybrid Models*	MLAL	0.258	0.970	0.146	0.706	0.187
			MLDA	0.203	0.984	0.129	0.560	0.207
	Test	*Single Model*	MLP	0.217	0.939	0.158	0.602	0.243
		*Hybrid Models*	MLAL	0.206	0.956	0.157	0.563	0.218
			MLDA	0.167	0.970	0.135	0.467	0.212
Tmax	Train	*Single Model*	MLP	0.584	0.955	0.376	1.610	0.224
		*Hybrid Models*	MLAL	0.466	0.968	0.313	1.290	0.199
			MLDA	0.404	0.983	0.240	1.119	0.170
	Test	*Single Model*	MLP	0.838	0.935	0.513	2.297	0.283
		*Hybrid Models*	MLAL	0.694	0.953	0.492	1.916	0.274
			MLDA	0.606	0.969	0.436	1.676	0.266
AUC	Train	*Single Model*	MLP	0.264	0.958	0.169	0.731	0.210
		*Hybrid Models*	MLAL	0.225	0.972	0.131	0.623	0.158
			MLDA	0.162	0.987	0.106	0.449	0.148
	Test	*Single Model*	MLP	0.218	0.942	0.176	0.600	0.240
		*Hybrid Models*	MLAL	0.187	0.959	0.145	0.518	0.198
			MLDA	0.137	0.975	0.108	0.384	0.177

Overall, MLDA achieved the strongest test performance across the three pharmacokinetic endpoints. Compared with the baseline MLPR, the test RMSE decreased from 0.217 to 0.167 for Cmax (23.0% reduction), from 0.838 to 0.606 for Tmax (27.7% reduction), and from 0.218 to 0.137 for AUC (37.2% reduction). The corresponding *R*^2^ values increased from 0.939 to 0.970 for Cmax, from 0.935 to 0.969 for Tmax, and from 0.942 to 0.975 for AUC.

### 4.3 Taylor diagram comparison of baseline and optimized models across PK targets

Figure 2 compares the predictive behavior of the baseline MLP with the optimizer-enhanced hybrid models (MLAL and MLDA) using Taylor diagrams by integrating correlation, standard deviation, and centered RMS error to assess the extent to which each model reproduces the statistical features of the observed pharmacokinetic data. Regarding Cmax, both hybrid models are closer to the reference point than the baseline MLP. MLDA is the closest to the measured standard deviation arc and also shows the highest correlation coefficient, thus indicating that the variability in peak concentration has been better preserved, and the RMS deviation is lower. The observed pattern is consistent with the lower error metrics presented in Table 6. With respect to Tmax, the variation of the models is larger, thus it is more reflective of the biological variability of the timing-related PK parameters. MLDA still keeps the highest correlation and a shorter radial distance than the baseline model, thereby indicating that the variability in the absorption timing has been handled better. MLAL also makes a better performance than MLP; however, it shows a slightly greater deviation in standard deviation. Regarding AUC, the models are close together near the reference point; however, MLDA is the closest to the ideal combination of correlation and variance once again. This implies that the total drug exposure is reconstructed more accurately.

**Figure 2:**
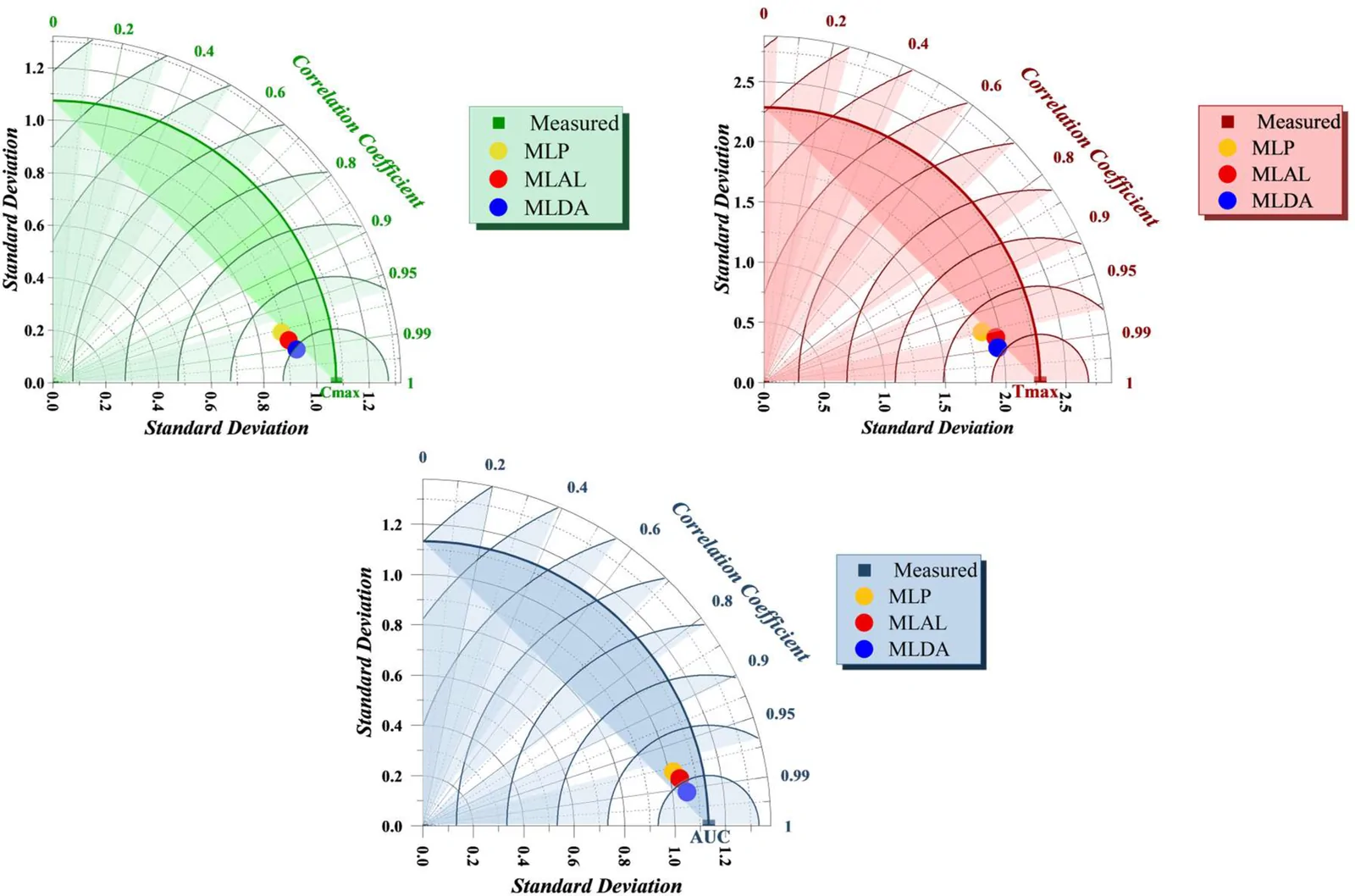
Taylor diagram illustrating the correlation and standard deviation of the models relative to the observed data, offering a comprehensive assessment of their performance.

### 4.4 Distributional error analysis demonstrating variance reduction and generalization strength

Figure 3 assesses the strength and the ability to generalize of the predictive models by looking at the full distribution of the percentage errors for Cmax, Tmax, and AUC, thus providing the information beyond the usual point metrics. Such a distribution-level evaluation is crucial since pharmacokinetic predictions rely not only on average accuracy but also on the stability of the model and the capacity to refrain from large deviations that may affect clinical decisions. In all three PK endpoints, the hybrid models show significantly tighter and more centered error distributions during training; thus, they demonstrate less variance and a lower number of large-magnitude deviations. MLDA has the most concentrated profiles, which means that it has stronger control over the prediction noise and better suppression of outliers—a scenario in which such an effect is especially useful in parameters like Tmax, where biological variability usually leads to larger errors. MLAL also raises the level of the baseline MLP; however, its wider tails show that there is still some variability left. During the test stage, the overall spread of errors is larger than in the training phase, which is a common occurrence with new data; however, MLDA still keeps the distributions that are the narrowest and the most symmetrical. This pattern shows that the generalization of the model is better and that there is no overfitting, as the extension of the reduction of the extreme errors is consistent across Cmax, Tmax, and AUC. These distributional shifts correspond to the numerical changes seen in Table 6; thus, they are a confirmation that the optimizer-guided calibration leads to not only better average accuracy but also to the increased trustworthiness of single predictions. This less widened error range has, in fact, some practical implications for pharmacokinetic modeling: the more precise Cmax predictions provide the safety of dosing within exposure limits, the better Tmax estimates allow the accurate timing of the therapeutic onset, and the stably predicted AUC gives the system a way to evaluate the exposure.

**Figure 3:**
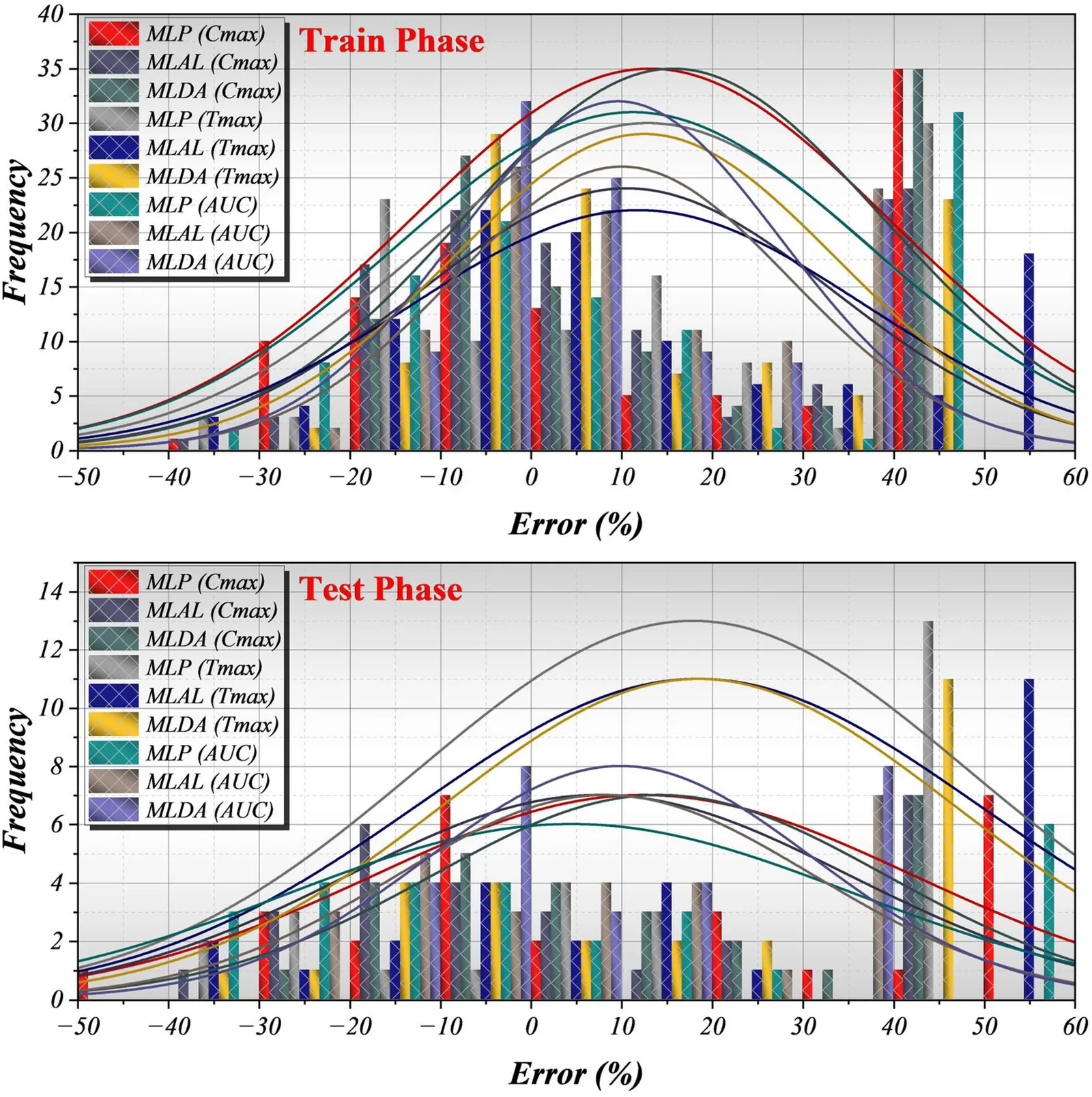
Histogram for visualizing the distribution of prediction errors across different models, offering a clear overview of their error patterns and comparative behavior.

### 4.5 Tri-objective Pareto analysis of accuracy, interpretability, and computational cost

Figure 4 brings together accuracy, explanatory strength, and computational cost in one Pareto space to assess how the MLDA model balances trade-offs of these three competing objectives across the three pharmacokinetic endpoints. Such a multi-objective perspective is necessary since PK prediction is hardly based on a single metric; its reliable implementation depends on the efficient balancing of error reduction, interpretability, and computational practicality. The figure makes visible that the optimization landscape for each target is very different. Cmax covers the most favorable region of the Pareto space, thus suggesting that MLDA achieves a good balance of low RMSE and high *R*^2^ with a small computational burden. AUC reveals a similarly favorable trade-off, albeit with slightly higher cost, which is in line with the generally broader dynamic range that is usually associated with exposure-related metrics. Conversely, Tmax is the farthest from the Pareto-optimal region, thus reflecting the physiological variability in absorption timing as well as the modeling difficulty that has been referred to in the PK literature. One of the main novelties of this analysis is that it captures tri-objective behavior, performance patterns that cannot be inferred from RMSE or *R*^2^ alone. By identifying trade-offs for each endpoint, the figure makes it possible to understand that MLDA responds differently to each PK target and not that it optimizes them uniformly.

**Figure 4:**
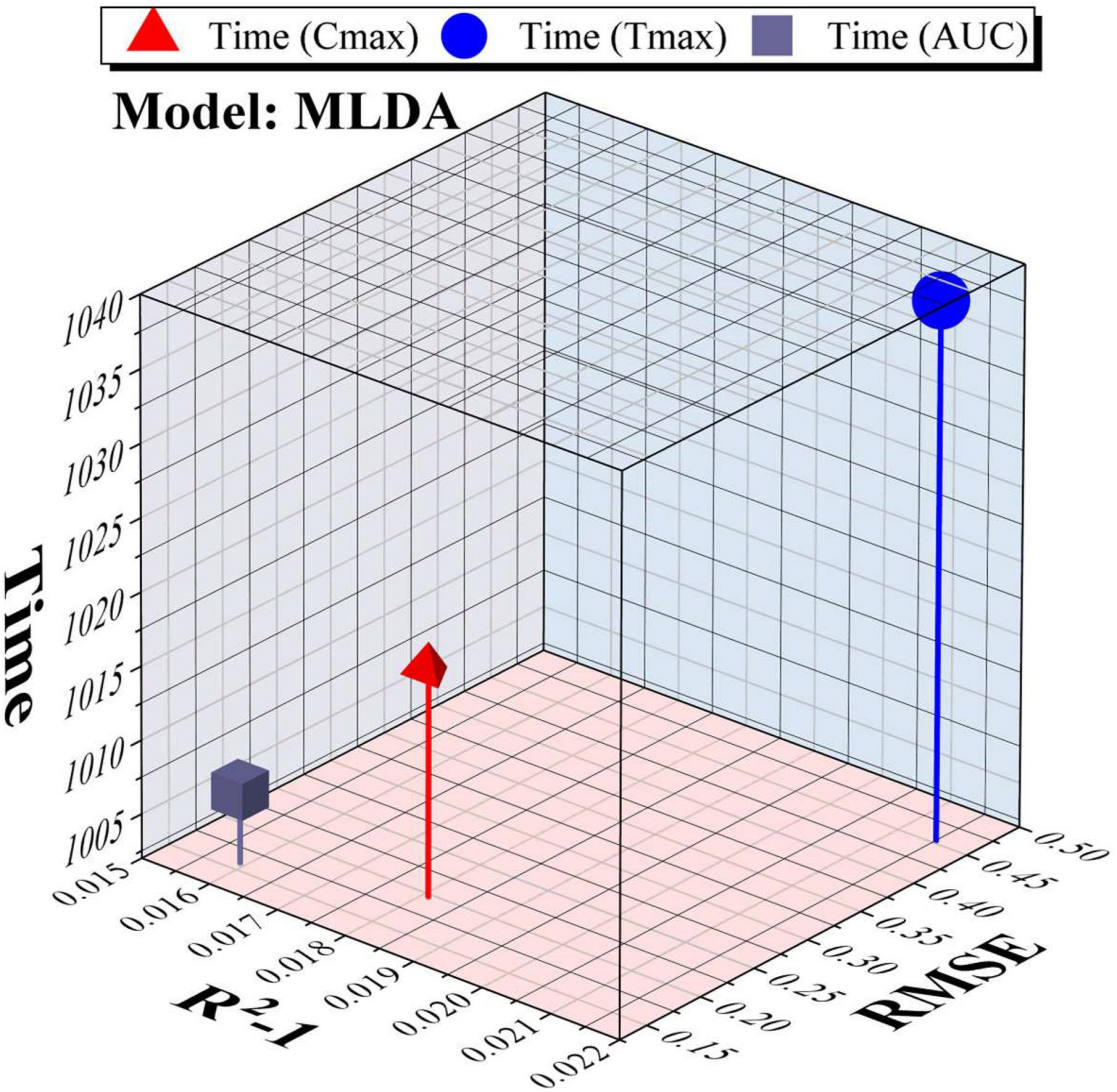
3D Symbol plot for visualizing the Pareto front of the best model for each target, providing an overall view of their comparative performance.

### 4.6 Friedman *post-hoc* significance map comparing optimizers across PK prediction tasks

Figure 5 shows the pairwise Friedman *post-hoc p*-values for the three hybrid MLPR models over the pharmacokinetic targets Cmax, Tmax, and AUC. Each heatmap summarizes how the DAOA, ALO, and OOA differ in their rank distributions after K-fold evaluation, thus giving a comparative view of model behavior rather than absolute performance. From a statistical point of view, the Cmax panel consistently presents very low *p*-values, thus pointing to significant differences in the rankings of all pairs of models. On the contrary, the Tmax findings display a mixed pattern: in some cases, the 0.05 threshold is exceeded, thus indicating similar rankings, while in other cases, the *p*-values remain significant. As for AUC, all *p*-values are high, thus indicating that there are no statistically significant differences among the three optimizer-guided models. The results show that the choice of the Optimizer affects the model ranking, where the ranking of the model is stable. From the scientific point of view, these patterns demonstrate how the differently targeted optimization strategies lead to changes in the prediction aspects of pharmacokinetics. The distinct separations in Cmax rankings imply that the prediction of peak concentration is highly dependent on how the Optimizer explores the hyperparameter landscape, which is very important in modeling exposure-related toxicity. The partial divergence in Tmax rankings indicates the moderate sensitivity in capturing absorption timing. The strong agreement between different optimizers for AUC shows that the model is robust in predicting the total drug exposure, which is a very good property that facilitates reproducibility and reliability within the proposed multi-optimizer nanocarrier modeling framework. Friedman ranking analysis was applied to repeated model performance observations obtained under identical cross-validation partitions and evaluation settings, thereby satisfying the repeated-measures comparison structure required for nonparametric rank-based testing.

**Figure 5:**
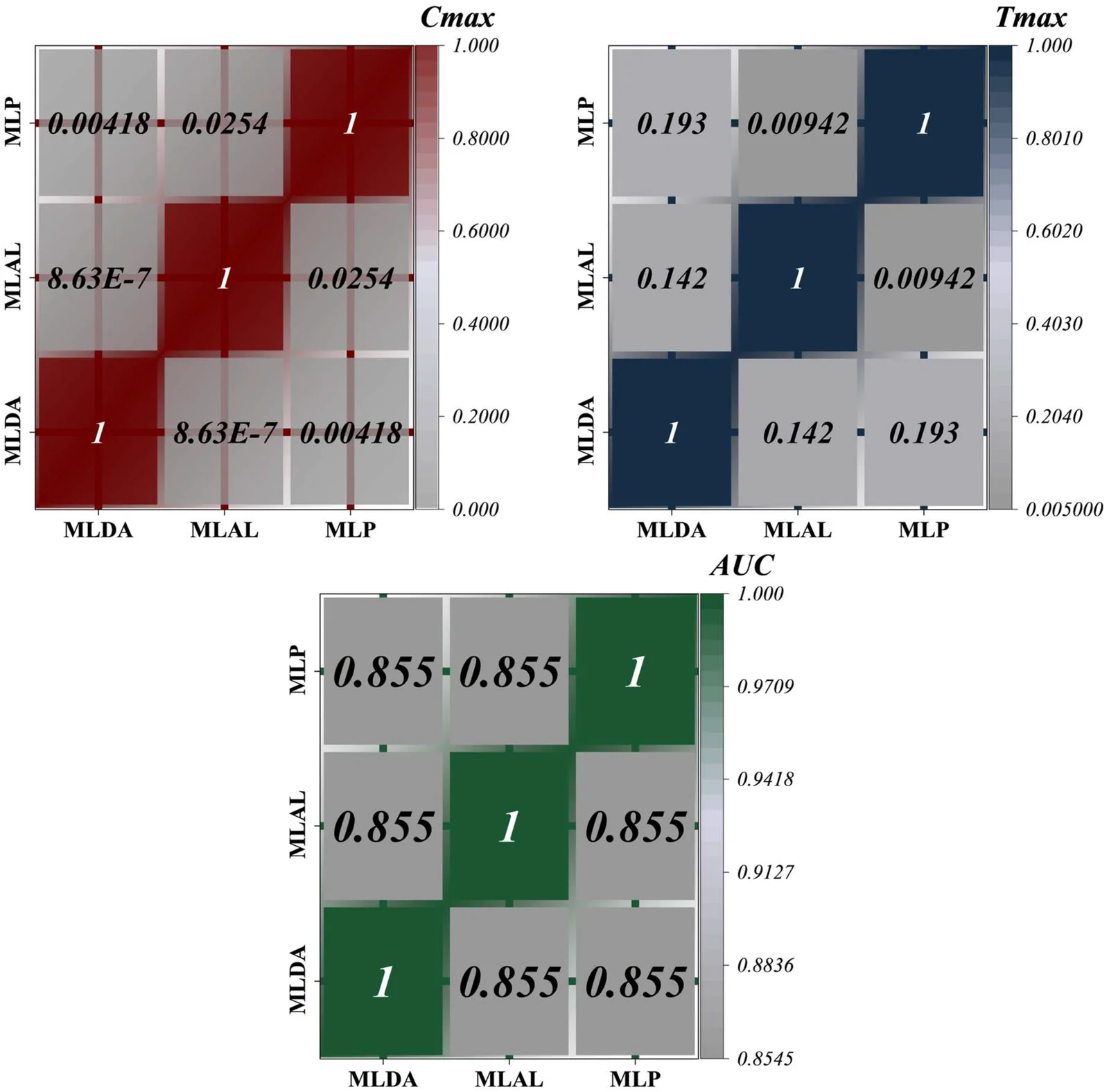
Heatmap plot for illustrating the results of the Friedman test, highlighting the distribution and comparative differences among models.

### 4.7 Computational runtime profiling for baseline and optimizer-enhanced MLP models

Figure 6 compares the computational runtime of the baseline MLPR and the optimizer-enhanced models for predicting Cmax, Tmax, and AUC. The runtime values are presented as relative computational observations obtained under identical local execution conditions and should not be interpreted as universal benchmarking results. The baseline MLPR completed training and evaluation in less than one second, whereas MLDA required approximately 1,000–1,038 s and MLAL required approximately 1,331–1,358 s. This substantial increase in computational time is expected because the optimized models require repeated population-based hyperparameter searches and multiple evaluations of candidate network configurations. The results therefore demonstrate a clear practical trade-off. Evolutionary optimization improved predictive performance but required considerably greater computational resources than the baseline model. Consequently, the optimizer-enhanced framework may be most appropriate when improved predictive accuracy justifies the additional computational cost, whereas the baseline MLPR may remain preferable when rapid model development is the primary requirement.

**Figure 6:**
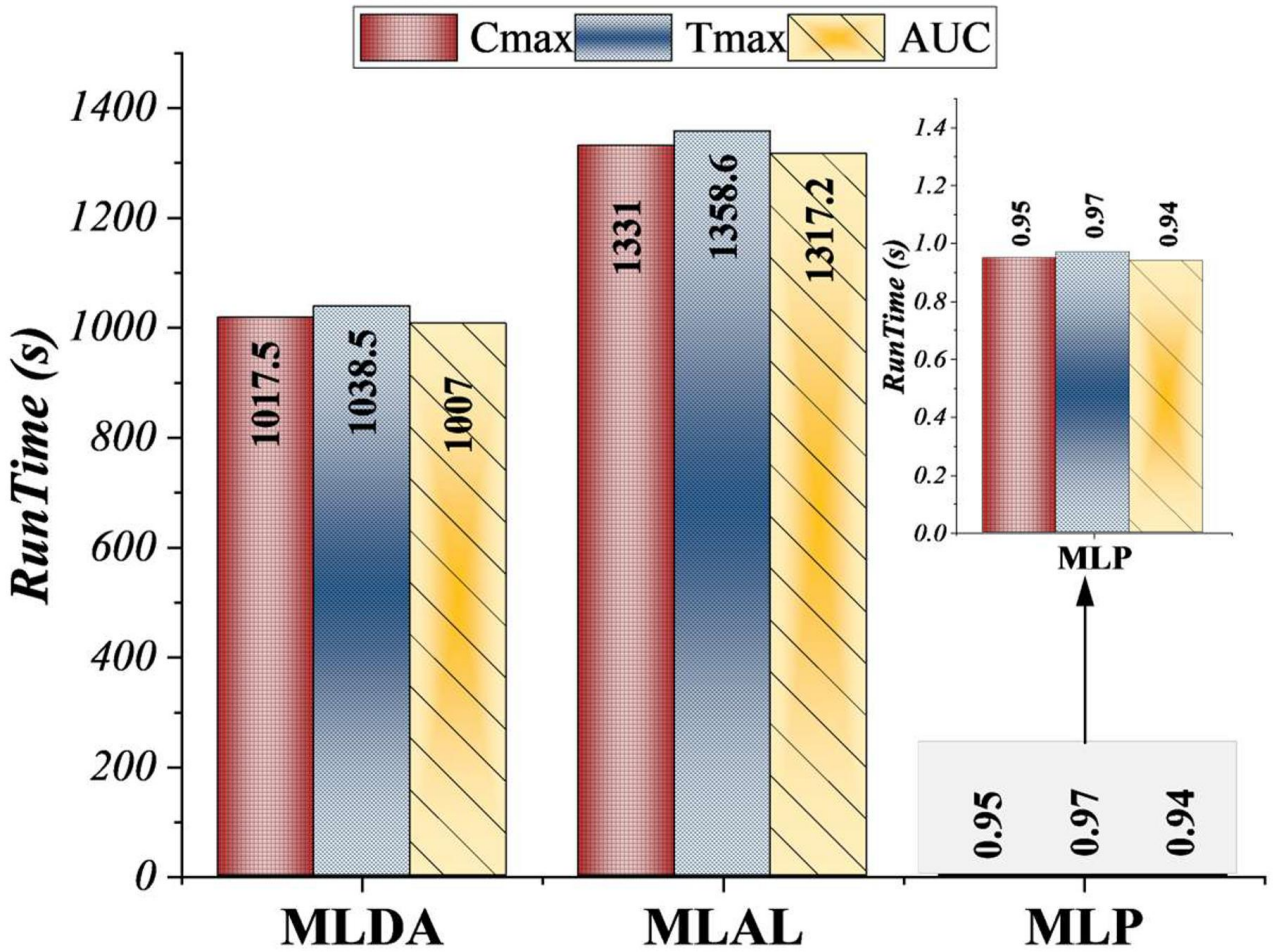
Column plot showing the computation runtime of each model during training and evaluation, providing a comparative view of their efficiency.

### 4.8 Prediction-interval bootstrapping and uncertainty visualization for PK test samples

Figure 7 visualizes the PIB values of the test samples referring to the three pharmacokinetic targets (Cmax, Tmax, and AUC) along with their 95% prediction intervals (lower and upper). The PIB sequences predicted for single samples are displayed in each subplot together with the uncertainty bounds obtained by the prediction-interval bootstrapping. The width of the intervals and the variability of the predictions differ markedly for the targets. In the case of Cmax, the intervals are relatively narrow and almost uniform, while the prediction line closely follows the bounds, thus indicating stable behavior at the sample level. Tmax reveals variations of PIB values to a great extent and also significantly broader prediction intervals of many samples, thus depicting not only the uncertainty of the model at the level of the data but also the higher variance of the time-dependent pharmacokinetic measurements usually inherent in nature. Oscillations in predicted values for AUC are of moderate magnitude, with interval widths mostly being at the same level as the central trend, thus showing that there is a balanced uncertainty around the cumulative exposure predictions. From a scientific point of view, these interval profiles emphasize the usefulness of incorporating uncertainty quantification together with point predictions. To provide prediction intervals specific to a sample makes the work more understandable, thus helping the judgment of the reliability of the different endpoints and showing where the variability is expected, especially for Tmax. The comparatively greater complexity of Tmax prediction may also reflect latent physiological variables absent from the current feature matrix. Factors such as circadian modulation of absorption, mucociliary clearance variability, epithelial permeability differences, transporter expression patterns, local membrane interactions, and biological clearance rhythms may influence the timing of peak concentration but are rarely standardized or consistently reported across published nanocarrier studies. Their absence may therefore contribute to residual predictive uncertainty for temporally sensitive pharmacokinetic endpoints.

**Figure 7:**
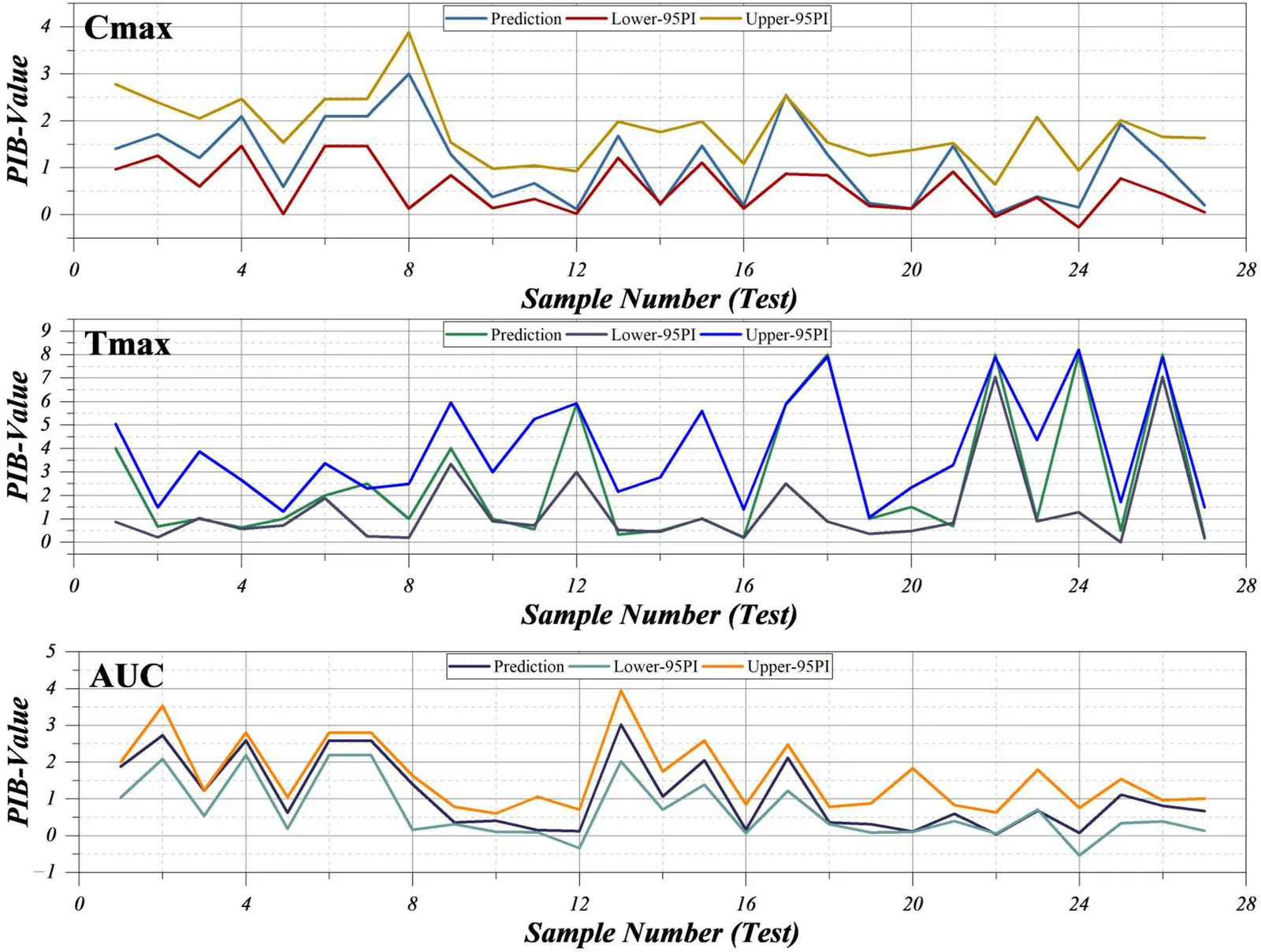
Line plot for the performance Index benchmark (PIB) of each feature for a given target, showing the predicted values along with the lower and upper 95% prediction intervals.

### 4.9 Interpretation of modelling outcomes and pharmacokinetic insights

Hybrid multi-optimizer MLPR architectures in this research are shown to be a significantly improved scenario for the prediction of nanocarrier pharmacokinetic behavior, especially for Cmax and AUC, when compared to conventional or non-optimized models. The major methodological advancement is the combination of evolutionary search with interaction-sensitive neural regressors, which not only results in higher accuracy of prediction but also facilitates structured uncertainty quantification, features that are hardly present in the ML field of nanomedicine. The results suggest that the PK relationships of nanocarriers are nonlinear and are influenced by multivariate interactions. Particle size was retained as a central nanocarrier descriptor because it strongly influences mucosal transport, systemic circulation, cellular uptake, release kinetics, and tissue penetration. Previous studies have demonstrated that nanoparticle size modulates biodistribution efficiency, blood-brain barrier interaction, and nasal retention characteristics, thereby directly affecting pharmacokinetic endpoints such as Cmax, Tmax, and AUC (44). From the mechanistic standpoint, the major factors of formulation weight, solubility, and drug-carrier ratio affecting Cmax are consistent with short-term absorption and dissolution kinetics, whereas the dependence of Tmax on particle size, surface charge, and hydrophobicity points to the diffusional transport that is delayed and typical of particulate systems. AUC, being an integrated exposure metric, is sensitive to the wider physicochemical variability, which is in line with heterogeneous disposition pathways. Compared with exposure-related endpoints such as Cmax and AUC, Tmax prediction presents a greater modeling challenge because it is governed by temporally dynamic mechanisms including drug release rate, carrier degradation, mucosal retention, tissue permeation delays, and biological transport kinetics. Unlike concentration- or exposure-based outputs, Tmax is particularly sensitive to small variations in absorption timing and formulation disintegration behavior. Although the current framework incorporated several physicochemical and formulation descriptors, certain temporally informative variables such as release-profile curvature, degradation half-life, residence duration, and absorption rate constants were not consistently reported across source studies and therefore could not be included in the integrated dataset. Future studies incorporating such descriptors may further stabilize Tmax prediction and improve temporal resolution.

Principal component analysis further supports this by showing that highly dispersed descriptors like size and weight dominate the global variance, while solubility and logP contribute localized, interaction-dependent effects. The enhanced performance of MLDA over MLAL and baseline MLP models can be explained by its better convergence dynamics and more thorough exploration of high-dimensional hyperparameter space. While this may increase the computational cost, the consequent stability, decreased error dispersion, and more narrow bootstrapped prediction intervals are indicative of a significant accuracy-efficiency trade-off. The uncertainty quantification component adds to the interpretability by, for example, differentiating endpoints that are predictably (Cmax) from those that are inherently variable (Tmax). The present results are in accordance with previous findings, which have indicated that PK prediction in nanocarrier systems is highly influenced by nonlinear interactions and dataset heterogeneity. However, the main advantage of the proposed approach is that it combines multi-objective optimization with probabilistic reliability assessment. Limitations are dataset heterogeneity, finite sample size, and the computational expense of evolutionary tuning. However, the architecture is extendable and represents a solid base for formulation design, virtual screening, and preclinical PK estimation of the next-generation nanocarrier platforms.

### 4.10 Feature importance interpretation

Principal component analysis and model sensitivity trends indicate that several descriptors consistently contributed to predictive behavior across pharmacokinetic endpoints. Among these, particle size, molecular weight, lipophilicity (logP), drug-carrier ratio, release characteristics, and selected structural composition variables showed strong influence on model response behavior. Particle size is mechanistically linked to permeability, circulation behavior, and uptake kinetics, while molecular descriptors influence solubility, membrane interaction, and distribution behavior. Although PCA-based interpretation provides insight into variable structure and variance contribution, future work should incorporate *post hoc* explainability techniques such as SHAP or LIME to quantify both global and local feature contributions in nonlinear prediction settings (Table 7).

**Table 7:** Key influential descriptors identified through PCA and model behavior analysis.

Feature	Mechanistic relevance
Particle size	Permeability, uptake, circulation, BBB interaction
Molecular weight	Diffusion and transport behavior
logP	Membrane permeability and lipophilic partitioning
Drug-carrier ratio	Loading and release kinetics
Release behavior	Temporal absorption and exposure
Composition variables	Structural interaction and stability

### 4.11 Practical implications and translational relevance

Results from this research show that the combination of multi-optimizer evolutionary search with MLPR represents a feasible computational avenue to significantly speed up the development of nanocarrier formulations. As the framework allows for the dependable prediction of Cmax, Tmax, and AUC directly from physicochemical descriptors, it is instrumental in the staged pre-screening of candidate designs even before their synthesis in the lab. The experimental workload is therefore reduced, the optimization cycles are shorter, and the material costs decrease, in particular, for formulations that are complex or are made of components of high value.

The use of bootstrapped prediction intervals is a preclinical decision-making tool that has a well-defined operational mode. By indicating the level of uncertainty around the PK estimates, the model enables researchers to differentiate those formulations that exhibit stable and predictable behavior from those that require further investigation. In this way, the study makes better early go/no-go assessments. These uncertainty-aware predictions also open the door for mechanistic interpretability: the insights, like the effect of particle size and hydrophobicity on Tmax, as well as the contribution of formulation weight in determining Cmax, can be used to guide the rationale for changes in dose and release characteristics during preclinical translation. The choice of Pareto-front analysis and reproducible optimization as the main focus of the methodology serves to create a standardized workflow that can be readily applied to datasets in nanomedicine. This, in turn, is extremely important since the field has been facing problems of heterogeneity and non-reproducible ML pipelines. The designed framework that encourages transparent, multi-objective model selection is also in line with the future regulatory demand for computational risk assessment and can, therefore, act as a support in forming industry settings for the justification of formulation strategies.

Beyond formulation optimization, the proposed framework may be incorporated into computer-aided pharmaceutical development pipelines for rapid virtual screening of candidate nanocarrier formulations prior to synthesis. The predictive model can support prioritization of promising formulations, estimation of expected pharmacokinetic performance, reduction of experimental iterations, optimization of formulation composition, and uncertainty-aware selection of candidates for subsequent animal studies. Such computational assistance may shorten formulation development cycles while improving resource allocation during early-stage pharmaceutical research (Figure 8).

**Figure 8:**
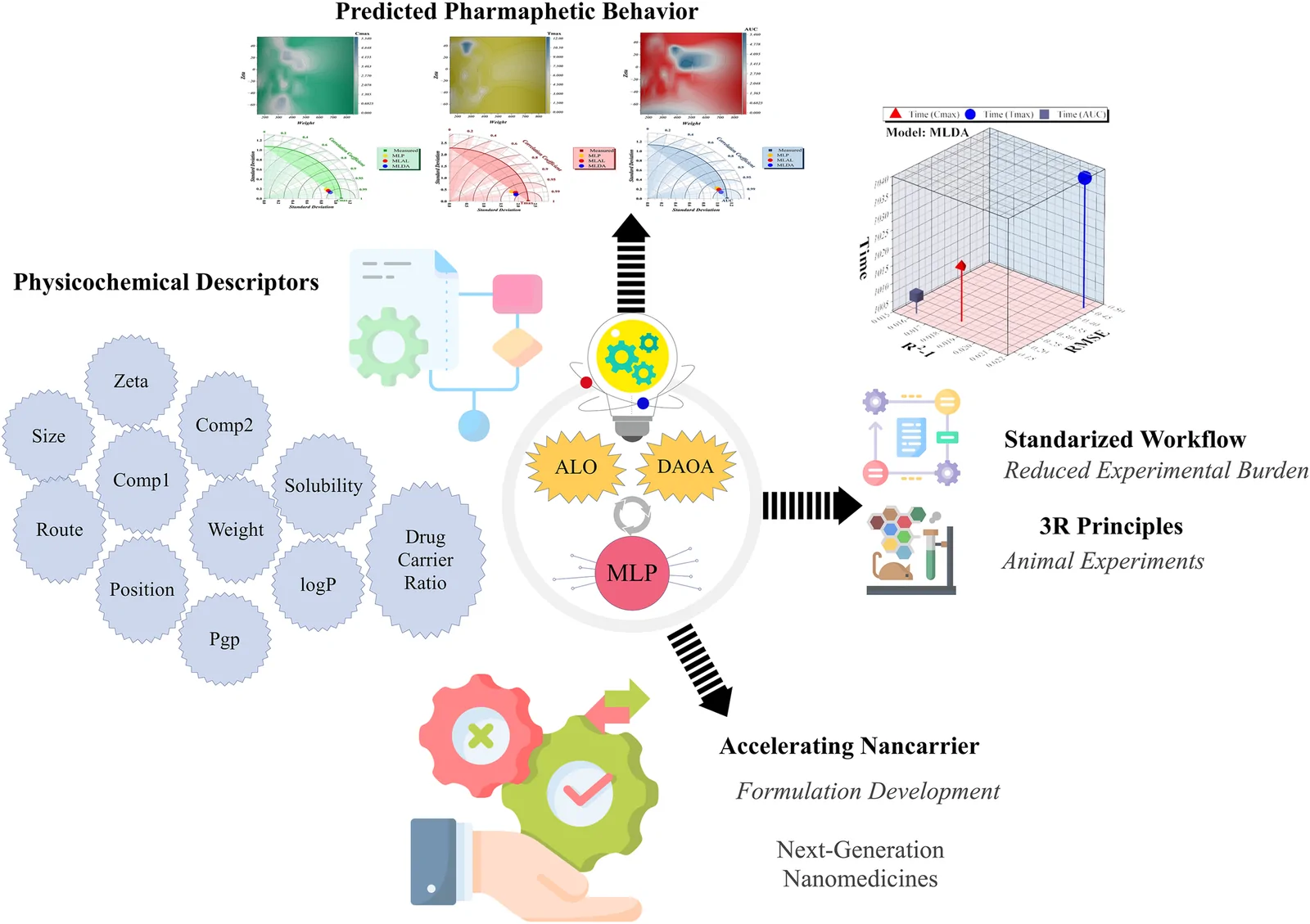
Practical implications of the proposed multi-optimizer MLPR framework.

### 4.12 Achievements, strengths, and limitations of the proposed framework

The proposed framework demonstrated several important achievements. First, it enabled direct prediction of pharmacokinetic endpoints from nanocarrier and molecular descriptors without requiring new experimental synthesis or *in vivo* testing. Second, the integration of literature-derived multi-study datasets improved formulation diversity and reduced dependence on single-study biases. Third, uncertainty quantification through bootstrapped prediction intervals provided an additional layer of prediction confidence often absent in conventional formulation models. The framework also offers several advantages, including strong nonlinear approximation capability, flexible optimizer-based hyperparameter tuning, and compatibility with heterogeneous descriptor spaces.

However, several limitations should be considered when interpreting the generalizability of the proposed framework. First, the literature-derived dataset contains cross-study heterogeneity associated with differences in experimental protocols, animal models, analytical procedures, formulation compositions, and pharmacokinetic measurement conditions. Although preprocessing and repeated cross-validation were applied to improve robustness, such heterogeneity may limit the direct transferability of the trained models to experimental settings that are substantially different from those represented in the current dataset. Second, the dataset contains a finite number of observations and may not fully represent the complete physicochemical and formulation design space of nanocarrier systems. Consequently, predictive performance may decrease when the framework is applied to formulations, drugs, carrier compositions, or biological conditions that are underrepresented in the available data. The absence of several potentially relevant biological and temporal descriptors may also contribute to residual uncertainty, particularly for Tmax prediction. Third, evolutionary hyperparameter optimization requires repeated evaluation of multiple candidate MLPR configurations and therefore introduces a substantial computational burden. This limitation may become more important when larger datasets, additional pharmacokinetic endpoints, or more complex predictive architectures are considered. The practical applicability of the optimization framework should therefore be evaluated according to the balance between the expected improvement in predictive performance and the available computational resources.

Although repeated five-fold cross-validation, optimizer stability assessment, and bootstrap-based uncertainty quantification were used to reduce the risk of overfitting, these procedures represent internal validation strategies. Independent external validation using standardized and prospectively generated nanocarrier datasets remains necessary to establish the real-world transferability and predictive generalizability of the proposed framework.

#### 4.12.1 Future research directions

Several opportunities exist for extending the proposed predictive framework. First, validation using completely independent prospective pharmacokinetic datasets should be conducted to further establish model generalizability under real-world experimental conditions. Second, incorporation of additional biological descriptors, including degradation kinetics, protein corona characteristics, biodistribution parameters, transporter expression, release-profile dynamics, and temporal absorption variables, may further improve prediction of complex pharmacokinetic endpoints such as Tmax. Third, future studies should benchmark the framework against additional machine learning architectures, including gradient boosting methods, transformer-based models, graph neural networks, and hybrid ensemble approaches. Fourth, explainable artificial intelligence techniques such as SHAP, LIME, and integrated gradients should be incorporated to improve mechanistic interpretation of learned relationships. Finally, prospective experimental validation using newly synthesized nanocarrier formulations would provide the strongest evidence of translational applicability and predictive reliability.

### 4.13 Comparative positioning against existing predictive models

Existing research in nanocarrier and BBB-targeted drug delivery has predominantly focused on mechanistic strategies for improving brain penetration, including polymeric carriers, lipid-based systems, ligand-functionalized nanoparticles, stem-cell-mediated delivery, and alternative administration pathways such as intranasal transport. Although these studies provide critical biological and formulation insights, comparatively fewer investigations have addressed quantitative pharmacokinetic prediction using integrated multi-study datasets. Recent advances in machine learning-assisted formulation modeling have demonstrated the utility of nonlinear predictive algorithms for complex physicochemical systems, including ensemble learners, support vector machines, neural networks, and optimization-assisted regression models. In the present study, multilayer perceptron regression was selected because nanocarrier pharmacokinetics involve highly nonlinear interactions among molecular descriptors, carrier characteristics, release behavior, and administration variables that are often poorly captured by linear or shallow parametric models. The integration of evolutionary hyperparameter optimization further improved robustness by enabling adaptive architecture tuning under heterogeneous cross-study conditions. Nevertheless, future work should benchmark the present framework against additional architectures such as gradient boosting methods, random forests, support vector regression, and hybrid ensemble models to further evaluate predictive generalizability and domain transferability. As shown in Table 8, prior studies have largely emphasized mechanistic transport enhancement or isolated formulation optimization, whereas the present work focuses on quantitative prediction of pharmacokinetic endpoints from integrated literature-derived nanocarrier descriptors. Future research should expand this framework by integrating larger multi-route datasets, external validation cohorts, richer temporal descriptors, mechanistic biological variables, and benchmarking against ensemble and hybrid machine learning architectures.

**Table 8:** Comparison of existing studies and present work.

Study category	Primary focus	Modeling type	Output
BBB mechanistic reviews	Biological transport mechanisms	Conceptual/mechanistic	BBB transport understanding
Nanocarrier optimization studies	Formulation enhancement	Experimental optimization	Improved delivery efficiency
Recent ML formulation studies	Property prediction	ML/optimization models	Physicochemical prediction
Present study	PK endpoint prediction from integrated nanocarrier descriptors	Optimized MLP framework with uncertainty quantification	Cmax, Tmax, AUC prediction

The scope of the present work was not to benchmark all machine learning architectures, but to evaluate the influence of different evolutionary optimization strategies on a unified nonlinear predictive framework. Multilayer perceptron regression was selected because pharmacokinetic behavior in nanocarrier systems is governed by complex nonlinear interactions among molecular, physicochemical, and formulation descriptors. The use of a common predictive architecture enabled controlled assessment of optimizer behavior, architecture tuning efficiency, and robustness under heterogeneous literature-derived data conditions. This design isolates the contribution of optimization strategy while avoiding confounding effects introduced by simultaneous architecture changes.

## 5 Conclusion

This study demonstrates the influence of nonlinear and heterogeneous interactions on the pharmacokinetics of nanocarriers, thereby indicating that these cannot be adequately represented by standard modeling approaches. The suggested multi-optimizer MLPR framework manages to uncover these intricate patterns, thus making significant progress towards the prediction of PK endpoint variables. To emphasize, the hybrid-optimized architectures recorded RMSE decreases by more than 20%–30% for Cmax and AUC in comparison with the baseline MLP, thus they were able to retain the *R*^2^ values above 0.85, which was stable, for repeated K-fold validation. Meanwhile, Tmax was characterized with higher variability and thus *R*^2^ values were usually noted to be within the range of 0.55–0.65, which indicates its higher physiological stochasticity. This research is set apart by several methodological contributions. This is an integrated predictive framework that combines DA, ALO, and OOA in a single MLPR architecture and, thus, allows multi-objective hyperparameter optimization instead of fixed or gradient-based tuning. The evolutionary search always led to the identification of architectures that exhibited smoother convergence behavior and were less prone to local minima, which was supported by the significant decrease in the U95 dispersion across all PK endpoints. The implementation of bootstrapped prediction intervals also allowed for the provision of calibrated uncertainty estimates, where average PI widths of ±8%–12% for Cmax and ±10%–15% for AUC were observed, thus enabling decision-making to be reliable. Computational cost vs. accuracy trade-off was another benefit supplied by the Pareto-front analysis, which provided a transparent mechanism for this purpose. A mechanistic explanation of the learned PK surfaces carries the biological plausibility of the results. Particle size and hydrophobicity were the main factors affecting Tmax, while formulation weight and solubility were the factors that had the strongest influence on Cmax.

## Data Availability

The original contributions presented in the study are included in the article/Supplementary Material, further inquiries can be directed to the corresponding author.
